# Multidimensional factors associated with ADHD core symptoms in children: cognition, sleep, behavior, and demographics

**DOI:** 10.3389/fpsyt.2025.1658202

**Published:** 2025-11-13

**Authors:** Yanhong Fu, Zixi Qin, Ling Qin, Hong Zhang, Hairun Liu, Siyan Huang, Dandan Li

**Affiliations:** 1 Center for Cognitive and Sleep, the People’s Hospital of Guangxi Zhuang Autonomous Region & Institute of Brain and Mental Diseases, Guangxi Academy of Medical Sciences, Nanning, China; 2 School of Computing and Information Technology, University of Wollongong, Wollongong, NSW, Australia

**Keywords:** ADHD, inattention, hyperactivity-impulsivity, cognitive function, sleep disturbances

## Abstract

**Objective:**

Attention-Deficit/Hyperactivity Disorder (ADHD) involves inattention (IA) and hyperactivity-impulsivity (HI), which may be linked to distinct developmental and behavioral profiles. This study examined the demographic, cognitive, sleep, and behavioral factors associated with IA and HI symptoms among children.

**Methods:**

A cross-sectional sample of 331 children aged 6–12 years (mean = 8.72 ± 1.45 years; 84.6% male), diagnosed with ADHD, participated in this study between December 2020 and December 2022. ADHD symptoms were assessed via Swanson, Nolan, and Pelham Rating Scale-IV (SNAP-IV); cognitive function with Chinese version of the Das-Naglieri Cognitive Assessment System (DN: CAS); sleep via the Sleep Disturbance Scale for Children (SDSC); and behavior using the Conners Parent Symptom Questionnaire (PSQ).

**Results:**

Regression analyses showed that HI symptoms were negatively associated with age (β = –0.197, *p* < 0.001) and higher in boys (β = –0.156, *p* = 0.004), while IA symptoms remained stable across age and gender. IA was significantly related to attentional deficits (β = –0.146, *p* = 0.037) and learning problems (β = 0.471, *p* < 0.001). HI showed stronger associations with conduct problems (β = 0.411, *p* < 0.001), sleep-wake disturbances (β = 0.164, *p* = 0.003), and low anxiety (β = –0.121, *p* = 0.033).

**Conclusion:**

These findings support dimension-specific understanding and targeted management of ADHD symptoms.

## Introduction

Attention Deficit Hyperactivity Disorder (ADHD) is a common neurodevelopmental disorder characterized by symptoms of inattention and hyperactivity-impulsivity, affecting approximately 5%-7% of school-aged children globally (1, 2). Regional variations exist, with approximately 10% prevalence in the United States (3) and 6.4% in China (4). Despite clear diagnostic criteria (DSM-5, ICD-11), ADHD treatment depends on parent-reported symptoms, and their remission is key to better function and quality of life (5). However, significant symptom heterogeneity complicates diagnosis and treatment, necessitating investigations into underlying influencing factors.

Both the executive function hypothesis and the cognitive processing theory posit that cognitive functional deficits are considered the core impairments of ADHD, with cognitive deficits potentially serving as endophenotypes of ADHD. A recent systematic review identified consistent executive function (EF) impairments in children with neurodevelopmental disorders, particularly in attention, response inhibition, working memory, and planning (6). These EF deficits correlate with ADHD core symptoms. Emerging evidence suggests symptom dimension–specific neural substrates: inattention is linked to deficits in attentional control, whereas hyperactivity-impulsivity is associated with impaired response inhibition (7). Complementing the EF framework, the cognitive processing theory proposes that disruptions in distinct cognitive operations—such as planning, attention, and successive processing—underlie ADHD pathology. Empirical studies using cognitive processing assessments have confirmed these deficits in children with ADHD (8, 9). However, the direct relationships between cognitive processing components and specific ADHD symptom dimensions remain less thoroughly explored than EF-based models. Clarifying these associations may deepen our understanding of the distinct cognitive-neural mechanisms underlying inattention and hyperactivity-impulsivity.

Comorbidities, notably behavioral issues and sleep disorders, frequently co-occur with ADHD, complicating clinical presentations and potentially exacerbating ADHD symptom severity. Approximately 50%-90% of children with ADHD exhibit at least one comorbid condition, such as learning disabilities, oppositional defiant disorder (ODD), or anxiety disorders (4, 10, 11). Additionally, prevalent sleep disturbances like difficulty initiating and maintaining sleep, excessive daytime somnolence, and sleep-wake transition disorders significantly correlate with worsened ADHD symptoms (12–15). These issues highlight the importance of comprehensively understanding how cognitive, behavioral, and sleep-related factors collectively influence ADHD symptom profiles.

However, critical gaps remain regarding the interactive roles of demographic characteristics, cognitive functioning, sleep disturbances (when considered as specific phenotypes rather than a unitary construct), and behavioral comorbidities in shaping ADHD symptomatology. In particular, the differential effects of discrete sleep phenotypes (e.g., disorders of initiating and maintaining sleep [DIMS], disorders of excessive somnolence [DOES], and sleep–wake transition disorders [SWTD]) and the modulatory role of emotional profiles—such as the reported inverse association between anxiety and hyperactivity-impulsivity—have been understudied. Addressing these gaps is pivotal for advancing precision diagnostics and developing tailored interventions. Therefore, in the present study we adopt a multidimensional framework that integrates demographic, cognitive (DN: CAS), sleep (SDSC subscales), and behavioral (Conners PSQ) measures to examine predictors of inattention and hyperactivity-impulsivity, with particular attention to specific sleep phenotypes and the low-anxiety/high-impulsivity relationship. This focused, integrative approach aims to clarify symptom-specific pathways and inform more individualized assessment and intervention strategies.

## Materials and methods

### Study design

This cross-sectional study was conducted at the outpatient Cognitive Center of Guangxi Zhuang Autonomous Region People’s Hospital, between December 2020 and December 2022. The research protocol was approved by the Ethics Committee of the People’s Hospital of Guangxi Zhuang Autonomous Region (Approval No.: KY-ZC-2017-33).

### Participants

Children aged 6 to 12 years who presented to the clinic during the study period were screened for eligibility. Inclusion criteria were: (1) diagnosis of ADHD based on the Diagnostic and Statistical Manual of Mental Disorders, Fifth Edition (DSM-5), confirmed by two attending psychiatrists using the Kiddie Schedule for Affective Disorders and Schizophrenia (K-SADS); (2) currently enrolled in elementary school (grades 1–6); (3) no use of ADHD medication in the past month; (4) full-scale IQ ≥70, assessed by the Chinese Wechsler Intelligence Scale for Children-Revised (WISC-CR) (16), and (5) absence of comorbid neurological or psychiatric disorders (e.g., autism spectrum disorder, epilepsy, schizophrenia), severe medical conditions (e.g., genetic syndromes, endocrine disorders), or current use of psychotropic medications. Exclusion criteria during the study included unwillingness to complete all assessments, withdrawal of consent, or loss to follow-up.

### Measurement tools

ADHD core symptoms were assessed using the Swanson, Nolan, and Pelham Rating Scale-IV (SNAP-IV), a validated 18-item parent-report questionnaire evaluating inattention and hyperactivity-impulsivity symptoms. Each item is rated on a 4-point Likert scale (0 = not at all, 3 = very much), with higher mean scores reflecting greater symptom severity (17).

Sleep disturbances were measured using the Sleep Disturbance Scale for Children (SDSC), a 26-item parent-report tool that evaluates six domains: disorders of initiating and maintaining sleep (DIMS), sleep breathing disorders (SDB), sleep–wake transition disorders (SWTD), disorders of arousal (DA), disorders of excessive somnolence (DOES), and nocturnal hyperhidrosis (SHY). Each item is rated on a 5-point Likert scale, and higher scores indicate more severe disturbances (18, 19).

Behavioral problems were assessed using the Conners Parent Symptom Questionnaire (PSQ), which includes subscales for conduct problems, learning problems, psychosomatic symptoms, and anxiety. Items are rated on a 4-point Likert scale (0 = never, 3 = frequently), with higher scores indicating greater behavioral difficulties (20).

Cognitive function was evaluated using the Chinese version of the Das-Naglieri Cognitive Assessment System (DN: CAS), which is based on the PASS theory and measures four domains (P: planning, SIM: simultaneous processing, AIT: attention, and SUS: successive processing). Each domain consists of three tasks. Raw scores were converted into standard scores (M = 100, SD = 15), with lower scores indicating greater cognitive impairment (21).

IQ was assessed using the WISC-CR, which yields Verbal IQ, Performance IQ, and Full-Scale IQ scores. Only children with Full-Scale IQ ≥70 were included (16).

### Outcomes

The primary outcomes were the severity of ADHD core symptoms, including inattention and hyperactivity-impulsivity, as measured by the mean scores on the corresponding subscales of the SNAP-IV questionnaire. These scores were used as continuous dependent variables in correlation and regression analyses.

### Statistical analysis

All statistical analyses were performed using SPSS 23.0 and Zstats software. Non-normally distributed continuous variables were reported as median with interquartile range (IQR). Spearman correlation analysis was used to explore associations among ADHD symptoms, cognitive function, sleep disturbance phenotypes, and behavioral problems. To assess the independent and cumulative effects of demographic, cognitive, sleep, and behavioral factors on ADHD symptoms, hierarchical multiple regression analyses were conducted. In each regression, inattention and hyperactivity-impulsivity symptoms were entered separately as dependent variables. Model 1 included demographic variables (gender and age); Model 2 added cognitive processing scores (planning, attention, simultaneous, and successive processing); Model 3 included sleep variables (DIMS, SDB, SWTD, DA, DOES, SHY); and Model 4 added behavioral problems (conduct, learning, psychosomatic symptoms, anxiety). Statistical significance was set at p < 0.05.

## Results

### Demographic and clinical characteristics

A total of 331 children diagnosed with ADHD were included in the final analysis. The mean age was 8.72 ± 1.45 years (range: 6–12), and the majority were male (n = 280, 84.6%). On the SNAP-IV, the median total score across all 18 items was 1.61 (IQR: 1.28–2.00). Inattention symptoms were more severe than hyperactivity-impulsivity symptoms, with median scores of 1.78 (1.44–2.33) and 1.44 (1.00–2.00), respectively. For cognitive function assessed via the DN: CAS, the median standardized scores were highest for simultaneous processing [106 (98–116)], followed by successive processing [102 (92–112)], attention [84 (77–92)], and planning [77 (69–83)]. When comparing the median of the mean factor scores across the six SDSC subscales, DIMS and SHY showed the highest mean median scores [2.00 (1.71–2.57) and 2.00 (1.50–3.00), respectively], followed by SWTD [1.67 (1.33–2.00)], DOES [1.60 (1.20–2.20)], SDB [1.33 (1.00–1.67)], and DA [1.00 (1.00–1.33)]. Behavioral problems assessed using the PSQ revealed that learning problems had the highest median severity score [1.50 (1.25–2.00)], followed by conduct problems [0.75 (0.58–1.25)], anxiety [0.25 (0.00–0.75)], and psychosomatic symptoms 0.20(0.00-0.40) (**Table 1**).

**Table 1:** Characteristics of all participants.

Variables	Min	Max	M25	M50	M75
Gender (F/M)	51/280				
Age	6.2	12.9	7.50	8.50	9.67
Cognition					
P	53	113	69.00	77.00	83
SIM	76	150	98.00	106.00	116
AIT	53	117	77.00	84.00	92
SUS	69	129	92.00	102.00	112
Sleep					
DIMS	7	29	12.00	14.00	18
SDB	3	12	3.00	4.00	5
SWTD	6	23	8.00	10.00	12
DA	3	9	3.00	3.00	4
DOES	5	20	6.00	8.00	11
SHY	2	9	3.00	4.00	6
Behavior					
Conduct problems	0	3	0.58	0.75	1.25
Learning problems	0	3	1.25	1.50	2
Psychosomatic symptoms	0	1.2	0.00	0.20	0.4
Anxiety	0	2.25	0.00	0.25	0.75
ADHD core symptoms					
IA	0.33	3	1.44	1.78	2.33
HI	0	3	1.00	1.44	2

IA, inattention; HI, hyperactive/impulsivity; P, planning; SIM, simultaneous processing; AIT, attention; SUS, successive processing; DIMS, disorders in initiating and maintaining sleep; SDB, sleep breathing disorders; SWTD, sleep-wake transition disorders; DA, disorders of arousal; DOES, disorders of excessive somnolence; SHY, nocturnal hyperhidrosis.

### Correlation analysis

Spearman correlation analysis indicated that attention function, as measured by DN: CAS, was significantly negatively correlated with inattention symptoms (r = –0.114, p = 0.039), suggesting that lower attention capacity was associated with greater inattentive severity. All six SDSC sleep phenotypes were positively correlated with inattention (r = 0.113 to 0.266, all p < 0.05), with the strongest correlation observed for SWTD (r = 0.266, p < 0.001). For hyperactivity-impulsivity symptoms, significant positive correlations were observed with DIMS (r = 0.206, p < 0.001), SWTD (r = 0.276, p < 0.001), DOES (r = 0.134, p = 0.015), and SHY (r = 0.142, p = 0.010).

All four PSQ behavioral domains were significantly positively correlated with both ADHD symptom dimensions. For inattention, the strongest correlations were found with learning problems (r = 0.562, p < 0.001), followed by conduct problems (r = 0.385), anxiety (r = 0.304), and psychosomatic symptoms (r = 0.163) (all p < 0.01). For hyperactivity-impulsivity, the strongest correlation was with conduct problems (r = 0.479, p < 0.001), followed by learning problems (r = 0.321), psychosomatic symptoms (r = 0.191), and anxiety (r = 0.175) (all p < 0.01). Full correlation results are presented in **Table 2**.

**Table 2:** Correlations between cognitive function, sleep disturbances, behavior problems and ADHD core symptoms.

Core symptoms	P	SIM	AIT	SUS	DIMS	SDB	SWTD	DA	DOES	SHY	Conduct problems	Learning problems	Psychosomatic symptoms	Anxiety
IA	-0.03	-0.01	-0.11^*^	-0.07	0.26^**^	0.18^**^	0.27^**^	0.14^*^	0.30**	0.11^*^	0.39^**^	0.56^**^	0.16^**^	0.30^**^
HI	-0.02	-0.01	-0.03	0.03	0.21^**^	0.07	0.28^**^	0.07	0.13^*^	0.14^**^	0.48^**^	0.32^**^	0.19^**^	0.18^**^

IA, inattention; HI, hyperactive/impulsivity; P, planning; SIM, simultaneous processing; AIT, attention; SUS, successive processing; DIMS, disorders in initiating and maintaining sleep; SDB, sleep breathing disorders; SWTD, sleep-wake transition disorders; DA, disorders of arousal; DOES, disorders of excessive somnolence; SHY, nocturnal hyperhidrosis. ^*^p<0.05, ^**^p<0.01.

### Hierarchical multiple regression analysis

Hierarchical regression was performed to identify predictors of ADHD core symptoms. For inattention, Model 1 included demographic variables, but neither gender (β = –0.014, p = 0.800) nor age (β = 0.015, p = 0.788) showed significant effects showed significant effects. In Model 2, the addition of cognitive variables revealed that attention (AIT) was a significant negative predictor (β = –0.146, p = 0.037), indicating that poorer attention performance was associated with more severe inattention. This effect remained significant in Model 3 (β = –0.134, p = 0.040), even after controlling for sleep disturbances. Notably, two sleep phenotypes also entered as significant predictors: DIMS (β = 0.149, p = 0.016) and DOES (β = 0.178, p = 0.003), suggesting that sleep initiation difficulties and excessive daytime sleepiness both contribute to inattentive symptoms. In the final model (Model 4), the addition of behavioral variables substantially improved model fit. Learning problems emerged as the most robust predictor of inattention (β = 0.471, p < 0.001), while AIT (β = –0.079, p = 0.160), DIMS (β = 0.107, p = 0.051), and DOES (β = 0.052, p = 0.332) lost statistical significance. This indicates that the relationship between sleep and cognitive factors with inattention may be partially mediated by behavioral learning difficulties (**Table 3**).

**Table 3:** Multivariate hierarchical regression analysis of the effects of gender, age, cognitive function, sleep, and behavioral problems on ADHD core symptoms.

IA							HI					
Variable	Unstandardized β coefficient	95.0% CI		SE	Standardized β coefficient	P value	Unstandardized β coefficient	95.0% CI		SE	Standardized β coefficient	P value
		lower limit	upper limit					lower limit	upper limit			
(constant)	1.31	0.53	2.10	0.40		0.00	2.19	1.28	3.11	0.46		0.00
Gender	-0.06	-0.21	0.09	0.08	-0.03	0.45	-0.24	-0.42	-0.07	0.09	-0.13	0.01
Age	-0.03	-0.07	0.01	0.02	-0.07	0.14	-0.11	-0.16	-0.06	0.02	-0.23	0.00
P	0.00	-0.01	0.01	0.00	0.02	0.78	0.00	-0.01	0.01	0.00	0.00	0.96
SIM	0.00	-0.01	0.00	0.00	-0.01	0.90	0.00	-0.01	0.00	0.00	-0.03	0.55
AIT	-0.01	-0.01	0.00	0.00	-0.08	0.16	0.00	-0.01	0.01	0.00	-0.04	0.48
SUS	0.00	-0.01	0.00	0.00	-0.04	0.40	0.00	-0.01	0.00	0.00	-0.05	0.36
DIMS	0.01	0.00	0.03	0.01	0.11	0.05	0.01	-0.01	0.03	0.01	0.06	0.31
SDB	0.03	-0.01	0.06	0.02	0.08	0.12	-0.01	-0.05	0.03	0.02	-0.04	0.48
SWTD	0.01	-0.01	0.03	0.01	0.05	0.37	0.04	0.01	0.07	0.01	0.16	0.00
DA	0.00	-0.06	0.06	0.03	0.00	0.96	-0.03	-0.09	0.05	0.04	-0.04	0.49
DOES	0.01	-0.01	0.03	0.01	0.05	0.33	0.00	-0.02	0.02	0.01	0.01	0.92
SHY	0.01	-0.02	0.04	0.01	0.04	0.37	0.02	-0.01	0.05	0.02	0.05	0.26
Conduct problems	0.11	-0.03	0.25	0.07	0.09	0.11	0.55	0.40	0.71	0.08	0.41	0.00
Learning problems	0.46	0.35	0.57	0.06	0.47	0.00	0.15	0.02	0.28	0.07	0.13	0.03
Psychosomatic symptoms	-0.01	-0.24	0.21	0.11	-0.01	0.90	0.25	-0.02	0.51	0.13	0.09	0.07
Anxiety	-0.02	-0.17	0.14	0.08	-0.01	0.84	-0.19	-0.37	-0.02	0.09	-0.12	0.03

IA, inattention; HI, hyperactive/impulsivity; P, planning; SIM, simultaneous processing; AIT, attention; SUS, successive processing; DIMS, disorders in initiating and maintaining sleep; SDB, sleep breathing disorders; SWTD, sleep-wake transition disorders; DA, disorders of arousal; DOES, disorders of excessive somnolence; SHY, nocturnal hyperhidrosis; CI, confidence interval; SE, standard error.

For hyperactivity-impulsivity, Model 1 showed that male gender (β = –0.156, p = 0.004) and younger age (β = –0.197, p < 0.001) were associated with greater symptom severity. These associations remained significant across all subsequent models. Cognitive variables introduced in Model 2 did not yield any significant predictors. In Model 3, DIMS (β = 0.144, p = 0.020) and SWTD (β = 0.220, p = 0.001) both emerged as significant sleep-related predictors. After incorporating behavioral variables in Model 4, conduct problems showed a strong and independent association with hyperactivity-impulsivity (β = 0.411, p < 0.001). SWTD (β = 0.164, p = 0.003), anxiety (β =-0.121, p = 0.033) and learning problems (β =0.132, p = 0.026) also remained significant, while the effect of DIMS attenuated (β = 0.056, p = 0.313). These findings indicate that both externalizing behaviors and sleep-related characteristics—particularly those involving sleep–wake transitions—as well as internalizing symptoms such as anxiety, contribute to the manifestation of hyperactive-impulsive symptoms in ADHD.

## Discussion

This study identified distinct predictors for the two core dimensions of ADHD. Hyperactivity-impulsivity symptoms decreased with age and were more pronounced in boys, whereas inattention symptoms remained stable and showed no gender difference. Inattention was primarily associated with attentional deficits and learning problems, while hyperactivity-impulsivity was more strongly linked to oppositional behaviors, sleep-wake transition disturbances, learning problems, and low anxiety levels, suggesting symptom-specific developmental and behavioral pathways. Importantly, the novelty of our work lies in its multidimensional, phenotype-focused approach: we simultaneously assessed demographic, cognitive, behavioral, and discrete sleep phenotypes, and explicitly examined the counterintuitive association between lower anxiety and greater hyperactivity-impulsivity. This focused integration provides more specific evidence on symptom-dimension–specific correlates than studies that treat sleep or behavior as unitary constructs.

The developmental trends and gender differences observed in this study are consistent with previous findings. Hyperactivity-impulsivity symptoms declined with age, while inattention remained stable—mirroring longitudinal results from Holbrook et al. (22) and reinforcing that inattention may persist regardless of ADHD subtype (23). Boys exhibited higher levels of hyperactivity-impulsivity, with no significant sex difference in inattention, in line with meta-analytic evidence (24, 25). These patterns highlight the risk of underdiagnosing girls with inattentive presentations, as noted by Carucci et al. (26), and support calls for gender-sensitive screening focused on subtle cognitive disengagement. Beyond demographic patterns, our study extends prior work by identifying symptom-specific predictors. Attentional deficits assessed by DN: CAS were selectively associated with inattention, refining earlier general cognitive findings (8, 9). We also found that SWTD independently predicted hyperactivity-impulsivity, providing a more nuanced view of sleep phenotype effects (12, 13). Finally, our finding that low anxiety was linked to greater impulsivity supports previous suggestions that anxiety may suppress overt hyperactive behaviors (27), underscoring its modulatory role in ADHD symptom expression.

The findings that inattention symptoms were significantly associated with attentional dysfunction and learning problems, while hyperactivity-impulsivity symptoms were primarily predicted by oppositional behavior, specific sleep disturbances, and low anxiety, suggest distinct neuropsychological pathways underlying the two symptom dimensions of ADHD. Deficits in attentional function, as assessed by the DN: CAS, reflect impairments in selectively maintaining focus, inhibiting irrelevant stimuli, and allocating processing resources under cognitive load. The Attention subtest includes Expressive Attention, Number Detection, and Receptive Attention, collectively targeting interference control and vigilance (8). These deficits directly impact classroom learning and explain the strong association with academic difficulties. Prior studies have demonstrated that children with ADHD show persistent impairments in attention and planning, which disrupt information processing efficiency (9). In our findings, attentional performance predicted inattention but not hyperactivity, indicating that the two symptom clusters may involve non-overlapping cognitive substrates. Sleep disturbances also displayed symptom-specific associations. Although DIMS and DOES initially correlated with inattention, these effects diminished after accounting for behavioral comorbidities, suggesting possible mediation. In contrast, SWTD—which include phenomena such as sleep talking, nightmares, and myoclonic jerks—remained a significant predictor of hyperactivity-impulsivity even after full adjustment. SWTD reflects instability in arousal regulation during sleep, which may extend into the daytime as behavioral dysregulation and poor impulse control (13). Disrupted sleep continuity may impair prefrontal cortical functioning and lead to heightened emotional reactivity and motor overactivity, especially in young children with neurodevelopmental vulnerabilities.

Conduct problems emerged as the strongest behavioral predictor of hyperactivity-impulsivity. This is consistent with prior research indicating symptom overlap between ADHD-HI and ODD, where shared behavioral descriptors (e.g., “interrupts,” “argues,” “defies”) may reflect underlying deficits in emotional regulation and impulse inhibition (25). The significant independent contribution of ODD symptoms supports the hypothesis that HI and conduct problems may co-arise from prefrontal–limbic dysfunction and reinforce one another through negative behavioral-emotional feedback loops. An additional novel finding is the inverse relationship between anxiety and HI symptoms. Previous research suggests that anxiety may inhibit externalizing behaviors through heightened sensitivity to criticism or punishment (27). In contrast, children with low anxiety may exhibit under-responsiveness to threat cues and increased risk-taking. Animal models such as SHRSP rats demonstrate that reduced anxiety correlates with impulsive behavior, linked to alterations in hippocampal plasticity and serotonergic pathways. These mechanisms may help explain why low-anxiety children in our study displayed elevated HI symptoms, suggesting a potential “low-anxiety/high-impulsivity” ADHD subtype with distinctive neurobiological underpinnings and potentially lower responsiveness to standard psychostimulant treatments. Children with ADHD show deficits in multiple neural circuits, including frontostriatal dopaminergic loops, corticostriatothalamic pathways, frontocerebellar networks, and reward circuits. These abnormalities manifest as impairments in attention, cognitive control, timing, and reward processing. Collectively, these findings support a multi-pathway model in which distinct neural dysfunctions contribute to the heterogeneous clinical presentation of ADHD.

The dimensional dissociation of ADHD symptoms observed in this study suggests the need for tailored clinical strategies. Inattention, associated with cognitive and learning deficits, may benefit from cognitive training and academic support, while hyperactivity-impulsivity, linked to oppositional behaviors and sleep-wake transition disturbances, may require behavioral management, emotion regulation, and sleep-focused interventions. The inverse relationship between anxiety and impulsivity points to a potential low-anxiety, high-impulsivity subtype that may respond poorly to conventional stimulants. Moreover, the underrecognition of inattentive symptoms in girls highlights the need for gender-sensitive screening approaches to improve diagnostic accuracy and early intervention.

This study has several limitations. First, as a cross-sectional study, causal or directional inferences cannot be drawn; longitudinal designs are needed to clarify temporal relationships among cognition, sleep phenotypes, behavior, and symptom dimensions. Second, all symptom and comorbidity measures were parent−reported, which may introduce recall or reporter bias and could underdetect internalizing symptoms such as anxiety; future studies should incorporate multi−informant assessments (parent, teacher, and child self−report). Third, sleep assessment relied on the parent−rated SDSC, which—while practical for large samples—cannot capture physiological sleep parameters, prospective sleep–wake patterns, or circadian preference; we did not collect sleep diaries, actigraphy, or morningness–eveningness measures in this cohort. Fourth, we did not assess restless sleep or Restless Legs Syndrome (RLS) symptoms, which have been implicated in ADHD and may share dopaminergic mechanisms; targeted RLS screening should be included in future work. Fifth, although we statistically controlled for full−scale IQ in multivariable analyses to reduce confounding by intellectual functioning, interactions among IQ, attention, and learning problems are complex and warrant more detailed investigation, including stratified or mediation analyses. Taken together, future studies should use longitudinal designs with multi−informant ratings, objective sleep monitoring (e.g., actigraphy or polysomnography), prospective sleep diaries, circadian preference scales, and RLS assessment to better delineate causal pathways and to validate the symptom−dimension–specific associations reported here.

## Conclusion

This study highlights the multidimensional nature of ADHD and the distinct predictors associated with its core symptom dimensions. Inattention and hyperactivity-impulsivity exhibit divergent developmental patterns and neurobehavioral correlates, underscoring the need for symptom-specific assessment and intervention strategies. Targeting cognitive deficits, behavioral comorbidities, sleep disturbances, and anxiety profiles may enhance individualized treatment planning. Future longitudinal and multi-informant research is warranted to clarify causal mechanisms and optimize precision care in ADHD.

## Data Availability

The original contributions presented in the study are included in the article/supplementary material. Further inquiries can be directed to the corresponding author.
